# The Effects of Combined Exercise on Health-Related Fitness, Endotoxin, and Immune Function of Postmenopausal Women with Abdominal Obesity

**DOI:** 10.1155/2015/830567

**Published:** 2015-05-14

**Authors:** Sung-Mo Park, Yi-Sub Kwak, Jin-Goo Ji

**Affiliations:** Department of Physical Education, Dong-Eui University, 995 Eomgwangno, Busanjin-gu, Busan 614-714, Republic of Korea

## Abstract

This study was conducted to examine the effects of combined exercise on health-related fitness, endotoxin concentrations, and immune functions of postmenopausal women with abdominal obesity. 20 voluntary participants were recruited and they were randomly allocated to the combined exercise group (*n* = 10) or the control group (*n* = 10). Visceral obesity was defined as a visceral-to-subcutaneous fat ratio ≥0.4 based on computed tomography (CT) results. Body composition, exercise stress testing, fitness measurement, CT scan, and blood variables were analyzed to elucidate the effects of combined exercise. The SPSS Statistics 18.0 program was used to calculate means and standard deviations for all variables. Significant differences between the exercise group and control group were determined with 2-way ANOVA and paired * t*-tests. The exercise group's abdominal obesity was mitigated due to visceral fat reduction; grip strength, push-ups, and oxygen uptake per weight improved; and HDL-C and IgA level also increased, while TNF-*α*, CD14, and endotoxin levels decreased. Lowered TNF-*α* after exercise might have an important role in the obesity reduction. Therefore, we can conclude that combined exercise is effective in mitigating abdominal obesity, preventing metabolic diseases, and enhancing immune function.

## 1. Introduction

In Korea, 26.5% of the total female population is obese, and women in their 30s, 40s, and 50s account for 28%, 34.4%, and 37.4%, respectively, of the total obese female population. A sharp increase in the prevalence of obesity has been particularly seen with onset of menopause [1], a time in which women experience many metabolic changes from female hormone fluctuations. It has been reported that women are at higher risk for obesity during this period due to increases in body fat [2]. Postmenopausal women, in particular, often develop abdominal obesity without weight change, due to excessive accumulation of visceral fat and hormonal changes in the abdomen [3].

Increased physical activity through regular exercise is very effective for mitigating obesity and improving health-related fitness at all ages [4, 5]. Park et al. [6] reported that combined exercise, resistance exercise combined with aerobic training, is more effective for mitigating obesity than aerobic training alone.

Recently, it was reported that gut microbiota is closely involved with obesity onset [7] and that endotoxins residing in gut flora are also closely correlated with visceral fat increases [8]. It was also reported that endotoxins suppress immune functions by inducing inflammatory responses [9].

Endotoxins promote the activation of inflammatory responses by stimulating microphages to secrete proinflammatory cytokines, including tumor necrosis factor-*α* (TNF-*α*) [10]. During this process, endotoxins are induced to bind to CD14 on the microphage membrane [11]. The endotoxin receptor CD14 plays an important role in inflammatory and innate immune responses [12–14]. As obesity can increase endotoxin levels, causing inflammatory responses, obese women can in turn suppress immune function.

In this vein, various studies on controlling endotoxin levels and immune functions are being conducted, and interest in reducing endotoxins and improving immune functions through exercise is increasing [15, 16].

Kim et al. [17] reported that the largest immune function improvements resulted from combined exercise when aerobic, resistance, and combined exercises were compared. Ng et al. [18] reported that there were no changes in concentrations of serum endotoxin and TNF-*α* after a one-time 21 km road race. Sloan et al. [19] reported that when 61 study participants performed high- and moderate-intensity aerobic exercise for 30–40 min, 4 times a week for 12 weeks, only high-intensity exercise decreased the concentration of serum endotoxins.

Clearly, there have been many studies about an acute bout of exercise and aerobic exercise on physical activity and immune functions, but there is a lack of studies on endotoxin concentration changes after combined physical exercise. Moreover, while there are many studies on obesity and inflammation, as well as exercise and immune functions, there are hardly any studies on the interplay between endotoxins, health-related fitness, and immune functions of postmenopausal women with abdominal obesity.

Accordingly, this study was conducted to examine the effects of combined exercise on health-related fitness, endotoxin concentrations, and immune functions of postmenopausal women with abdominal obesity.

## 2. Study Methods

### 2.1. Participants

The participants in this study were naturally postmenopausal middle-aged women with abdominal obesity. Other selection criteria included a lack of regular exercise habits, no prior or present history of genital-related diseases, and not being under treatment for any internal or gynecological diseases. As we explained above, 20 voluntary participants were randomly allocated to the combined exercise group (*n* = 10) or the control group (*n* = 10). And this research was conducted ethically according to international standards.

Visceral obesity was defined as a visceral-to-subcutaneous fat ratio ≥ 0.4 based on computed tomography (CT) results.

The participants' physical characteristics are summarized in Table 1.

### 2.2. Test Methods

#### 2.2.1. Body Composition Examination

A body composition analyzer (VENUS-5.5, Korea) was used to measure the height, weight, % body fat, body fat, and lean body mass (LBM) before and after 12 weeks of exercise. Body mass index (BMI) was calculated using weight/height (kg/m^2^). Blood pressure was measured with a mercury sphygmomanometer (Hico, Japan) after participants were stabilized for 30 min.

#### 2.2.2. Exercise Stress Testing

Exercise stress testing was conducted before and after 12 weeks of exercise, using the Balke treadmill protocol. Maximal exercise stress was defined as the presence of any 2 of the following: (1) manifestation of steady state with oxygen intake not exceeding 150 mL/min and expected maximum heart rate (220-age); (2) respiratory exchange ratio (RER) ≥1.15; and (3) ratings of perceived exertion (RPE) ≥17 [20]. Oxygen intake and heart rate during exercise were analyzed with an automatic metabolic analyzer (Quarkb2, Cosmed, Italy).

#### 2.2.3. Combined Exercise Program

In the week prior to training, 1-repetition maximum (1RM) measurements were taken, and resistance equipment training and treadmill acclimation exercises were performed. The 1RM for resistance training was measured using the formula from the Korea Institute of Science and Technology [21]. In weeks 1–6, 60% of 1RM was used for 8–10 repetitions. In weeks 7–12, 70% 1RM was used for 10–12 repetitions. Each session lasted for 30 min 3 times a week. For aerobic training, exercise speed and heart rate based on the maximal exercise test were used to calculate the target heart rate (THR), equivalent to 40–75% of the heart rate reserve (HRR), (THR = HRR × intensity (%) + resting heart rate), after which the target exercise speed was determined with a regression equation each for 40 min 3 times a week for 12 weeks.

The exercise program is as shown in Table 2.

#### 2.2.4. Fitness Measurement

Left and right grip strengths were measured with a hand dynamometer (T.K.K. 5101). Back strength was measured with a back dynamometer (T.K.K. 5102). And also, total sit-ups and push-ups performed in 30 seconds were measured. Flexibility was measured using the sit-and-reach test. All measurements were taken twice, and the highest value was recorded.

#### 2.2.5. CT Scan

To measure abdominal fat, a CT scanner (Samsung GE Sytec 3000 I) scanned the transverse sections one above and one below umbilical level. The area's total abdominal fat volume was calculated by considering tissues with a Hounsfield number from −250 to −50 to be fat. Using abdominal and peritoneal spread as the boundary, the areas were divided into visceral fat tissue and subcutaneous fat tissue, with which the visceral fat to subcutaneous fat ratio was calculated.

#### 2.2.6. Blood Analysis

Before and after program, 15 mL of blood was drawn from the antecubital vein under fasting, stable conditions. Levels of total cholesterol (TC), triglycerides, high-density lipoprotein cholesterol (HDL-C), and low-density lipoprotein cholesterol (LDL-C) were analyzed enzymatically using an autoanalyzer (Hitachi 7600-110/7170 Analyzer, Tokyo, Japan). Levels of TNF-*α*, CD14, and immunoglobulins (IgA, IgG, and IgM) were analyzed using enzyme-linked immunosorbent assay (ELISA), flow cytometry, and immunoturbidimetry, respectively.

### 2.3. Data Processing

The SPSS Statistics 18.0 program was used to calculate means and standard deviations for all variables. Significant differences between the exercise and control groups were determined with 2-way ANOVA and paired *t*-tests. For independent correlation between changes in endotoxin and oxygen intake per weight, the correlation coefficient and regression coefficient of each were obtained. *p* < 0.05 was considered significant.

## 3. Results

### 3.1. Changes in Abdominal Fat

Body composition and abdominal fat before and after the exercise program are given in Table 3. In the exercise group, visceral fat and the visceral to subcutaneous fat ratio (both *p* < 0.01) decreased significantly compared to preprogram values. Visceral fat (*F* = 7.619, *p* < 0.01) and the visceral to subcutaneous fat ratio (*F* = 29.894, *p* < 0.001) changed significantly over time and between groups. No control group variables changed after the program.

### 3.2. Changes in Health-Related Fitness

Health-related fitness changes are given in Table 4. In the exercise group, a significant decrease was seen in postprogram % body fat (*p* < 0.05) compared to preprogram values. Grip strength (*p* < 0.05), push-ups (*p* < 0.05), and maximum oxygen intake per weight (*p* < 0.01) increased significantly. Moreover, % body fat (*F* = 4.844, *p* < 0.05), grip strength (*F* = 4.583,  *p* < 0.05), push-ups (*F* = 5.562, *p* < 0.05), and maximum oxygen intake per weight (*F* = 7.555, *p* < 0.01) changed significantly over time and between group. No control group variables changed after the program.

### 3.3. Changes in Serum Lipids, TNF-*α*, Immune Functions, CD14, and Endotoxin

Cholesterol, TNF-*α*, and C-reactive protein (CRP) level changes are given in Table 5. In the exercise group, HDL-C level increased significantly following the 12-week program (*p* < 0.05), whereas TNF-*α* level decreased significantly (*p* < 0.05). Moreover, HDL-C (*F* = 9.728,  *p* < 0.01) and TNF-*α* (*F* = 5.147, *p* < 0.05) levels changed significantly over time and between groups. No control group variables changed after the program.

Immune functions, CD14, and endotoxin values from before and after the program are given in Table 6. Values of IgA (*p* < 0.01) and IgG (*p* < 0.05) increased significantly after program, whereas CD14 (*p* < 0.05) and endotoxins (*p* < 0.01) decreased significantly. Moreover, IgA (*F* = 8.333, *p* < 0.01), CD14 (*F* = 5.434, *p* < 0.05), and endotoxin (*F* = 8.963, *p* < 0.01) levels changed significantly over time and between groups. No control group variables changed after the program.

## 4. Discussion

### 4.1. Effects on Abdominal Fat

Postmenopausal women often develop abdominal obesity without weight change from excessive accumulation of visceral fat in the abdomen [3], attributed to decreased lipolysis as a result of reduced estrogen [22]. As a result, many postmenopausal women experience decreased LBM and increased % body fat, most frequently in the abdominal area [23]. Postmenopausal women are also more at risk from obesity than premenopausal women [24]. That is, more severe abdominal obesity results in greater probability of metabolic disease development [25]. Therefore, prevention and mitigation of abdominal obesity in middle-aged postmenopausal women are important.

Kim et al. [26] reported that combined exercise and stretching (80 min sessions 5 times a week for 8 weeks) reduced visceral fat in middle-aged obese women. Park et al. [27] reported that an aerobic exercise regimen (60 min sessions 5 times a week for 12 weeks) reduced visceral fat in middle-aged men. Choi and Chun [5] also reported that circuit training (60 min sessions 4 times a week for 12 weeks) mitigated obesity.

In the present study, a 90 min combined exercise session (10 min warm-up, aerobic exercise for 40 minutes, resistance exercise for 30 min, 10 min cool-down) was performed 3 times a week for 12 weeks, resulting in reduced visceral fat (*p* < 0.01) and a reduced visceral to subcutaneous fat ratio (*p* < 0.001). In addition, a significant difference in results was seen between the control and exercise groups and over time, consistent with previous studies.

These results indicate that regularly performing combined exercise helps reduce visceral and subcutaneous abdominal fat in postmenopausal women. Combined exercise is thus useful for prevention and mitigation of obesity in this group.

### 4.2. Changes in Health-Related Fitness

The ACSM [28] separates motor skill-related fitness from health-related fitness and divides health-related fitness into body composition, flexibility, muscle strength, muscle endurance, and cardiovascular endurance. Decline in any of these elements' scan results in an increased likelihood of contracting a disease [29].

Health-related fitness is closely related to daily life. Leaner body composition decreases the probability of obesity and metabolic disease. Greater muscle endurance increases motor performance and decreases the probability of developing hypokinetic diseases. Flexibility can reduce muscle and joint injuries, and increased cardiovascular endurance reduces the probability of developing cardiovascular diseases [30]. Clearly, health-related fitness is very important in maintaining and improving the quality of daily life, a view supported by many studies. Kim et al. [31] reported that body fat, % body fat, and cardiorespiratory fitness improved in severely obese women as a result of a 12-week, low-intensity walking program. Shin et al. [32] reported that body fat, % body fat, muscle mass, cardiovascular endurance, muscle strength, and flexibility improved significantly in obese female college students as a result of dumbbell lifting and walking for 60 min sessions 4 times a week for 12 weeks.

Although exercise type and intensity influence areas of improvement, most previous studies reported that exercise improved overall health-related fitness. This study supported those findings. The 12-week combined exercise program reduced body fat and % body fat and improved cardiorespiratory fitness through aerobic exercise and also improved muscle strength and endurance by increasing LBM through resistance exercise. It is thus considered appropriate exercise to improve obese people's overall health-related fitness.

### 4.3. Changes in Serum Lipids and TNF-*α*

The functions and roles of serum lipid components occur mostly in the liver. They do not exist independently in the blood but are transported combined with proteins in particle form as “lipoproteins.” Lipoproteins are classified as HDL-C (transporting cholesterol from peripheral tissues to the liver for degradation) or LDL-C (accumulating cholesterol in the blood vessels) [33]. Obesity increases the prevalence of metabolic diseases, such as cardiovascular disease and insulin resistance, by raising these serum lipid levels, and HDL-C and LDL-C, in particular, are important indicators for prediction and evaluation of coronary artery diseases. Park et al. [34] reported significant increases in HDL-C and significant decreases in TC among serum lipids in middle-aged women with abdominal obesity after performing a combined exercise regimen of 60 min sessions 4 times a week for 12 weeks. Kang and Jung [35] also reported significant decreases in TC and significant increases in HDL-C in obese people after an aerobic exercise regimen of 40-minute sessions 4 times a week for 12 weeks.

In the present study, the exercise group's LDL-C, TC, and TG values decreased, while HDL-C values increased. Although Paek [36] reported that there were no significant differences in middle-aged women's TC values after performing aerobic dance 4 times a week for 12 weeks, most studies reported positive improvements in serum TC and TG levels as a result of regular training. In particular, HDL-C improvements, a cardiovascular risk factor, were seen in the present study. We believe that combined exercise can strengthen cardiopulmonary functions and help prevent and mitigate cardiovascular diseases and hyperlipidemia from HDL-C caused obesity.

Additionally, TNF-*α*, one of the inflammatory cytokines and a primary modulator of systematic response to infectious diseases, is closely related to obesity. Its serum concentrations are known to increase during high-intensity exercise, and high levels of TNF-*α* are secreted by adipose tissues [37].

Samartín and Chandra [38] reported that TNF-*α* and TNF-*α* gene activations, accompanied by weight loss, were observed in obese people. Ziccardi et al. [39] conducted a 1-year weight-loss program with 56 middle-aged obese women and saw a significant TNF-*α* decrease. Kondo et al. [40] reported significant decreases in TNF-*α* in obese women after 7 months of slope jogging and walking. This study's decrease in TNF-*α* levels was consistent with previous studies and is believed to result from reduced adipose tissue secretion of TNF-*α* after combined exercise decreased body fat, % body fat, and visceral fat.

### 4.4. Changes in Immune Functions, CD14, and Endotoxins

Immunoglobulins (IgA, IgG, and IgM) function as antibodies, carrying out humoral immune functions against antigens that invade the body [41].

IgA is found mostly in saliva and stops bacteria or microorganisms from invading the body by binding to them at sites vulnerable to bacterial invasion. IgG is the most abundant serum antibody. IgM is early antibody and is highly involved with bacteriolysis. The known normal values of immunoglobulins IgA, IgG, and IgM are 160–330 mg/dL, 700–1400 mg/dL, and 60–150 mg/dL, respectively [42].

Nehlsen-Cannarella et al. [43] reported that appropriate exercise increased the serum immunoglobulin concentrations, whereas Mackinnon [44] found no effect. Chun et al. [45] reported that IgM decreased significantly in middle-aged women following combined exercise for 12 weeks, and Tennyson and Friedman [7] reported significant increases in IgG in middle-aged obese women after 12 weeks of Nordic walking. In the present study, IgA increased significantly. In summary, based on this study and previous studies, it is believed that the immunoglobulins concentrations differ according to exercise time and duration, intensity, and participant age.

Endotoxins stimulate microphages by binding to CD14 on the microphage membrane. The stimulated microphages secrete proinflammatory cytokines, such as TNF-*α*, IL-6, and IL-1*β*, sometimes triggering excessive innate immune responses and causing a variety of immune responses and diseases, as well as aggravating existing diseases [9, 46]. In addition, when concentrations of serum endotoxin exceed 2 to 3 times the normal level, it becomes difficult to modulate the extent of inflammation, which induces weight gain [47]. In other words, endotoxins are believed to be important obesity-related and metabolic disease risk factors.

While studies on endotoxins and exercise responses are lacking, the present study showed that visceral fat and endotoxin decrease after a 12-week combined exercise program. Therefore, combined exercise is likely effective in reducing endotoxin concentrations. Lowered TNF-*α* after exercise might have an important role in the obesity reduction and increased immune function.

## 5. Conclusions

In the present study, middle-aged, postmenopausal women with abdominal obesity performed 90 minutes of combined aerobic and resistance exercises 3 times a week for 12 weeks in order to examine the effects of combined exercise on health-related fitness, endotoxin levels, and immune functions. The following conclusions were obtained.

We conclude that, as a result of the combined exercise program, abdominal fat, health-related fitness, immune response, and endotoxins all changed significantly over time and between groups. As seen in the above results, the exercise group's abdominal obesity was mitigated due to visceral fat reduction; grip strength, push-ups, and oxygen intake per weight improved; and HDL-C and IgA increased, while TNF-*α*, CD14, and endotoxin levels decreased.

Therefore, for middle-aged postmenopausal women with abdominal obesity, a 12-week combined exercise regimen improved % body fat and health-related fitness and increased immune function, while a decrease in visceral fat and endotoxin concentrations was seen. Accordingly, we believe that combined exercise is effective in mitigating abdominal obesity, preventing metabolic diseases, and enhancing immune function. Additionally, endotoxins may be a potential risk factor for obesity. Lowered TNF-*α* after exercise might have an important role in the obesity reduction and increased immune function.

In addition, making specific diet programs for postmenopausal woman to achieve a better control of variables on preventing metabolic diseases and mitigating obesity is urgently needed in the near future.

## Figures and Tables

**Table 1:** The characteristic subjects.

Variable	Exercise group (*n* = 10)	Control group (*n* = 10)
Age (yr)	57.20 ± 2.57	57.20 ± 1.69
Body weight (kg)	64.90 ± 3.83	64.76 ± 3.26
Height (cm)	157.95 ± 2.57	155.62 ± 3.14
Percent body fat (%)	34.32 ± 2.79	34.63 ± 1.60
BMI (kg/m^2^)	26.02 ± 1.55	26.80 ± 1.09

BMI: body mass index.

**Table 2:** Combined exercise programs.

Parameter	Event		Time
Warm-up	Head walk, neck stretch, shoulder circling, ribs extension, standing side raise, leg stretch, and triangle stretch		10 min

Aerobic exercise	Variety	Duration	40 min
	Running	1~6 weeks HRR 40~55%	
		7~12 weeks HRR 56~75%	

	Variety	Duration	
Resistance exercise	Arm curl, leg curl, leg extension, squat, chest press, low back, shoulder press, lat pulldown, back extension, and sit-up	1~6 weeks 1RM 60% 8–10 repetition × 3 sets	30 min
		7~12 weeks 1RM 70% 10–12 Lep × 3 sets	

Cool-down	Hand and leg shaking, stretch, and walking		10 min

**Table 3:** The changes of abdominal fat between the groups at baseline and after 12 weeks of training.

Variables	Group	Baseline	12 weeks	*F*-value
Total fat volume (cm^3^)	Exercise	741.61 ± 90.98	667.53 ± 84.64	G × P: 2.592
	Control	738.67 ± 87.86	753.41 ± 85.29	

Visceral fat volume (cm^3^)	Exercise	238.78 ± 31.69	198.27 ± 27.97^∗∗^	G × P: 7.619^##^
	Control	237.00 ± 30.90	248.78 ± 29.12	

Subcutaneous fat volume (cm^3^)	Exercise	502.84 ± 60.66	469.26 ± 57.49	G × P: 0.972
	Control	501.67 ± 57.70	504.63 ± 58.44	

V/S	Exercise	0.47 ± 0.02	0.42 ± 0.02^∗∗^	G × P: 29.894^###^
	Control	0.47 ± 0.02	0.49 ± 0.02	

G × P: group × period.

Significantly different from baseline: ^∗∗^ *p* < 0.01.

Significant interaction among groups and period: ^##^ *p* < 0.01, ^###^ *p* < 0.001.

**Table 4:** The changes of health-related fitness between the groups at baseline and after 12 weeks of training.

Variables	Group	Baseline	12 weeks	*F*-value
Body weight (kg)	Exercise	64.9 ± 3.83	62.5 ± 2.94	G × P: 2.112
	Control	64.7 ± 3.26	65.3 ± 3.36	

% Body fat (%)	Exercise	34.3 ± 2.78	31.4 ± 2.57^∗^	G × P: 4.844^#^
	Control	34.6 ± 1.59	34.8 ± 1.69	

Lean body mass (kg)	Exercise	41.0 ± 2.83	42.9 ± 2.47	G × P: 1.502
	Control	41.2 ± 3.53	40.7 ± 3.42	

Grip strength (kg)	Exercise	23.88 ± 2.97	27.40 ± 2.791^∗^	G × P: 4.583^#^
	Control	23.81 ± 3.21	23.35 ± 2.75	

Back strength (kg)	Exercise	49.38 ± 3.50	55.64 ± 10.96^∗^	G × P: 1.000
	Control	47.16 ± 11.61	45.85 ± 11.16	

Sit-up (frequency/30 sec)	Exercise	10.30 ± 5.51	13.60 ± 3.97	G × P: 1.740
	Control	9.00 ± 3.94	8.50 ± 4.60	

Push-up (frequency/30 sec)	Exercise	7.50 ± 2.75	11.90 ± 3.66^∗^	G × P: 5.562^#^
	Control	7.50 ± 3.37	7.10 ± 2.99	

Sit-and-reach (cm)	Exercise	12.21 ± 5.26	13.27 ± 5.33	G × P: 0.198
	Control	11.13 ± 4.17	10.86 ± 3.98	

Closed-eyes one-legged standing time (sec)	Exercise	9.22 ± 5.82	9.58 ± 3.75	G × P: 0.908
	Control	8.47 ± 5.95	8.37 ± 10.82	

VO_2_max (mL/kg/min)	Exercise	27.16 ± 3.40	31.75 ± 3.87^∗∗^	G × P: 7.555^##^
	Control	27.67 ± 3.68	26.89 ± 3.37	

G × P: group × period.

Significantly different from baseline: ^∗^ *p* < 0.05, ^∗∗^ *p* < 0.01.

Significant interaction among groups and period: ^#^ *p* < 0.05, ^##^ *p* < 0.01.

**Table 5:** The changes of serum lipids and TNF-*α* between the groups at baseline and after 12 weeks.

Variables	Group	Baseline	12 weeks	*F*-value
HDL-C (mg/dL)	Exercise	50.97 ± 3.86	55.74 ± 2.33^∗∗^	G × P: 9.728^##^
	Control	50.98 ± 3.53	49.10 ± 3.54	

LDL-C (mg/dL)	Exercise	148.20 ± 25.66	135.10 ± 16.96	G × P: 1.121
	Control	143.80 ± 28.73	146.60 ± 21.99	

TC (mg/dL)	Exercise	219.40 ± 36.73	192.30 ± 34.72	G × P: 1.566
	Control	216.50 ± 36.50	218.60 ± 39.46	

TG (mg/dL)	Exercise	137.10 ± 16.07	125.00 ± 11.03	G × P: 2.863
	Control	135.50 ± 15.93	138.80 ± 13.93	

TNF-*α* (pg/mL)	Exercise	2.32 ± 0.50	1.90 ± 0.05^∗^	G × P: 5.147^#^
	Control	2.32 ± 0.25	2.42 ± 0.24	

G × P: group × period.

Significantly different from baseline: ^∗^ *p* < 0.05, ^∗∗^ *p* < 0.01.

Significant interaction among groups and period: ^#^ *p* < 0.05, ^##^ *p* < 0.01.

HDL: high-density lipoprotein cholesterol, LDL: low-density lipoprotein cholesterol, TC: total cholesterol, TG: triglyceride, and TNF-*α*: tumor necrosis factor-*α*.

**Table 6:** The changes of immune function and CD14 and endotoxin between the groups at baseline and after 12 weeks.

Variables	Group	Baseline	12 weeks	*F*-value
IgA (mg/dL)	Exercise	211.70 ± 18.13	256.50 ± 48.75^∗∗^	G × P: 8.333^##^
	Control	217.80 ± 34.24	200.80 ± 26.55	

IgG (mg/dL)	Exercise	1365.10 ± 83.46	1376.80 ± 70.30	G × P: 0.791
	Control	1353.40 ± 89.20	1320.00 ± 76.47	

IgM (mg/dL)	Exercise	109.35 ± 20.98	112.78 ± 22.00	G × P: 0.542
	Control	109.60 ± 17.35	104.00 ± 16.67	

CD14	Exercise	5.84 ± 0.67	5.20 ± 0.57^∗^	G × P: 5.434^#^
	Control	5.99 ± 0.60	6.18 ± 0.34	

Endotoxin (EU/mL)	Exercise	0.33 ± 0.03	0.29 ± 0.03^∗∗^	G × P: 8.963^##^
	Control	0.33 ± 0.03	0.35 ± 0.03	

G × P: group × period.

Significantly different from baseline: ^∗^ *p* < 0.05, ^∗∗^ *p* < 0.01.

Significant interaction among groups and period: ^#^ *p* < 0.05, ^##^ *p* < 0.01.

CD14: cluster of differentiation 14, IgA: immune globulin A, IgG: immune globulin G, and IgM: immune globulin M.
